# Minimally Invasive and Navigation-Assisted Fracture Stabilization Following Traumatic Spinopelvic Dissociation

**DOI:** 10.3390/jcm14041289

**Published:** 2025-02-15

**Authors:** Mina Y. Girgis, Alex Tang, Michael S. Pheasant, Kenneth L. Koury, Michael T. Jung, Tan Chen

**Affiliations:** 1 Geisinger Orthopaedic Surgery Northeast Residency, Wilkes-Barre, PA 18711, USA; 2 Department of Orthopaedic Trauma Surgery, Geisinger Wyoming Valley Medical Center, Wilkes-Barre, PA 18711, USA; 3 Department of Orthopaedic Spine Surgery, Geisinger Medical Center, Danville, PA 18721, USA

**Keywords:** spinopelvic dissociation, pelvis, sacrum, minimally invasive spine surgery, lumbopelvic fixation

## Abstract

Spinopelvic dissociation is a highly unstable orthopedic injury with a growing incidence worldwide. Operative treatment classically involves an open lumbopelvic fusion and sacroiliac stabilization, which carries high perioperative morbidity and mortality in a frail patient population. Advancements in spinal navigation, robotics, and minimally invasive surgery (MIS) techniques now allow these fracture patterns to be treated entirely percutaneously through small incisions. These incisions are just large enough to accommodate pedicle screw guides and enable the placement of lumbopelvic instrumentation, with rods being passed subfascially across pedicle screws and extending caudally to iliac fixation. This contrasts with the open midline approach, which requires more extensive soft tissue dissection and results in increased blood loss compared to percutaneous techniques. Modern imaging techniques, including CT navigation and robotics, facilitate the precise placement of sacral S2AI screw instrumentation in both open and percutaneous methods, all while safely avoiding previously placed trans-sacral fixation and other existing hardware, such as acetabular screws. Trans-sacral screws are typically percutaneously inserted first by the orthopedic trauma service, utilizing inlet, outlet, and lateral sacral fluoroscopic guidance to navigate the limited available corridor. With the advent of MIS techniques, trauma patients can now benefit from faster postoperative rehabilitation, minimal blood loss, decreased pain, and quicker mobilization. This article will review current concepts on spinopelvic anatomy, fracture patterns, indications for treatment, and current concepts for minimally invasive percutaneous lumbopelvic fixation, and it will present illustrative examples.

## 1. Introduction

Sacral fractures overall follow a bimodal distribution, with young patients sustaining fractures following high-energy injuries and elderly patients often presenting after a low-energy fall from standing. Spinopelvic dissociation is a highly unstable orthopedic injury with an increasing global incidence, accounting for 3–5% of all sacral fractures [1]. Traditionally, the standard surgical treatment involved open lumbopelvic fusion combined with sacroiliac stabilization. However, this approach is associated with significant perioperative morbidity and mortality, particularly when this injury occurs in medically frail and geriatric patients following a low-energy mechanism [2,3,4,5]. However, most cases reported in the literature involve high-energy trauma, while the incidence of geriatric fragility-related spinopelvic dissociation fractures—the focus of this paper—remains poorly defined. Furthermore, challenges such as instrumentation failure, wound-related complications, and the substantial cost of revision fusion surgery, averaging USD 28,000 per case, highlight the need for alternative treatment strategies [6]. Consequently, the advancement of minimally invasive surgical (MIS) and navigation techniques offers a promising alternative to mitigate complications and the costs associated with open surgery, particularly in the geriatric population.

The advent of spinal navigation, robotics, and MIS techniques has revolutionized the management of these complex fracture patterns. These innovations allow for entirely percutaneous treatment through lumbopelvic instrumentation with subfascial rod passage between pedicle screws extending caudally to iliac fixation. Additionally, modern imaging technologies facilitate the safe placement of sacral instrumentation, even in cases involving prior trans-sacral fixation by orthopedic trauma surgeons. Furthermore, MIS approaches offer trauma patients numerous advantages, including decreased operative time, reduced blood loss, decreased postoperative pain, and quicker rehabilitation [7,8,9,10,11].

The primary objective of this article is to review key concepts of spinopelvic anatomy, fracture patterns, indications for treatment, and current advancements in minimally invasive percutaneous lumbopelvic fixation using illustrative examples to highlight these principles.

## 2. Anatomical Considerations and Surgical Indications

Spinopelvic fixation relies on a thorough understanding of the anatomical and biomechanical characteristics of the lumbar spine and pelvis. The transition from the relatively mobile lumbar spine to the rigid pelvis occurs at the spinopelvic junction, a key area of instability prone to stress risers leading to conditions such as spondylolisthesis [12]. Furthermore, the sacral spine poses unique challenges to fixation due to its predominantly cancellous bone composition compared to the denser cortical bone of the lumbar spine [12]. The sacral pedicles are shorter and wider, offering less cortical surface for fixation. Additionally, the sacral slope introduces significant vertical shearing forces between the lumbar and sacral segments, necessitating specialized stabilization strategies [12]. One critical biomechanical consideration in spinopelvic anatomy is the lumbosacral pivot point which is located at the dorsal L5-S1 intervertebral disc. Effective spinopelvic fixation must include stabilization that extends ventrally beyond this point to counteract mechanical stresses and maintain overall construct integrity (i.e., utilizing long screws that anchor into the ilium rather than being confined to the sacrum). However, anatomic variants such as sacral dysmorphism, which occurs in approximately 41% of patients, can make spinopelvic fixation more challenging and prone to screw misplacement [13]. A dysmorphic pelvis may also result in a corridor that is too narrow, making it unsuitable for trans-sacral screw placement [14]. In these cases, intra-operative navigation using computed tomography scans provides greater accuracy in avoiding screw misplacement compared to traditional two-dimensional imaging with fluoroscopy [15].

Unstable fracture patterns are a critical indication for spinopelvic fixation. Sacral fractures are classified according to the Denis system into three zones. Zone 1 involves the lateral sacral foramen, zone 2 passes through the foramen, and zone 3 is medial to the foramen. Zone 2 fractures can be further categorized by morphology, including H, U, λ, and T types, which share the destabilizing feature of horizontal and vertical fracture lines. These fracture patterns, classified as spinopelvic dissociation, disrupt the continuity between the axial spine and pelvis, often necessitating spinopelvic fusion in the form of triangular osteosynthesis to restore stability and prevent neurological compromise [16].

## 3. Natural History of Spinopelvic Dissociation

The natural history of spinopelvic dissociation carries a risk of worsening low back pain, ambulatory dysfunction, deformity and neurologic injury, particularly when kyphosis exceeds 20 degrees [17]. Given these potential risks, operative management is generally recommended. However, non-surgical treatment may be considered for non-ambulatory geriatric patients with multiple medical comorbidities deemed unfit for surgery. For these patients, non-operative treatment includes non-weightbearing of bilateral lower extremities and bedrest for approximately three months, which is required for fracture healing [17].

For patients who are otherwise expected to be ambulatory, non-operative treatment is associated with significant and often unnecessary morbidity and mortality, particularly in geriatric populations. Common complications include sacral decubitus ulcers, respiratory infections, and thrombotic events. The one-year mortality rate for bedbound geriatric patients is 15.2%, but it can rise to as high as 61.5% in those with three or more complications during their inpatient stay [18]. Therefore, surgical management is generally preferred in the appropriate surgical candidate, as it allows patients to initiate immediate weightbearing to avoid the high risk of complications associated with non-operative treatment.

## 4. Evolution of Open Surgery to Minimally Invasive Techniques for Lumbopelvic Fixation

Traditionally, displaced spinopelvic dissociation injuries have been treated via open spine surgery. However, open spinal surgery in patients with spinopelvic dissociation carries a high risk of morbidity and mortality with its extensive tissue dissection, increased blood loss and operative time [7,19]. Perioperative complications, including but not limited to deep surgical site infections, respiratory disorders, cardiac events, urinary tract infections, and delirium, can occur in up to 81.8% of this patient population following surgery [19]. Geriatric patients, who often also have a high prevalence of coronary artery disease, are especially vulnerable to the adverse effects of prolonged surgical times and extended hospital stays. These factors increase the risk of cardiac complications and delirium, both of which are associated with higher mortality rates [20,21]. Therefore, when a spinal fracture requires stabilization without the need for extensive decompression, MIS techniques can be utilized to minimize surgical risks and reduce morbidity. However, open techniques are sometimes necessary, particularly in cases of infected revisions that require thorough irrigation and debridement. Additionally, when there is significant neurological compression or compromise, an open approach may be necessary to allow for more extensive decompression and neural structure preservation.

Traditional iliac bolts have been the historic treatment to achieve distal fixation for spinopelvic constructs. This technique involves preparing the starting point medial and cephalad to the posterior superior iliac spine (PSIS) with a high-speed burr to attempt to mitigate hardware prominence and irritation. Bilateral screws are then inserted through the iliac corridor, with proper placement confirmed using iliac oblique and down-the-wing fluoroscopic views. These screws are connected to the L5 and S1 pedicle screws using iliac connectors and multi-axial clamps, restoring lumbar lordosis and achieving stability [16].

While this technique effectively stabilizes the spine to the pelvis in cases of spinopelvic instability, it has several drawbacks concerning the soft tissue envelope. The lateral starting point required additional muscle dissection away from the midline incision, increasing soft tissue disruption [6]. To address this, the screws were later adapted for placement using minimally invasive techniques. Despite this advancement, the lateral starting position continued to pose issues, including symptomatic hardware prominence and wound dehiscence, often necessitating reoperation [6].

A more modern technique involves the use of S2-Alar-Iliac (S2AI) screws, which offer a more medial starting point approximately 5 mm caudal and 2–3 mm lateral to the S1 foramen and is commonly placed in a minimally invasive fashion [6]. A high-speed burr is used to prepare the site, and screws are directed toward the greater trochanter, traversing dense cortical bone above the sciatic notch. This trajectory provides greater cortical purchase than iliac bolts. The alignment of S2AI screws with the lumbar construct eliminates the need for additional connectors, reduces soft tissue dissection, and minimizes screw prominence, making them particularly advantageous for minimizing wound complications and hardware failure [6,12,22,23,24].

Trans-sacral screws are another well-established, minimally invasive technique for stabilizing posterior pelvic ring fractures and have also been adapted for use in cases of spinopelvic dissociation with minimal displacement [25]. This adaptation emerged to mitigate the significant time demands and morbidity associated with open lumbopelvic fixation [25]. The lateral ilium serves as the entry point, with inlet and outlet pelvic fluoroscopic views guiding the anterior–posterior and cranial–caudal screw trajectories, respectively. Lateral sacral views further ensure safe placement within the bony corridor. Proper imaging and tactile feedback during screw insertion are critical for avoiding complications, such as bony breaches or neurologic injury.

Although trans-sacral screws offer the advantage of being minimally invasive, they are primarily used in minimally displaced fractures due to specific limitations. The proximal sacral segment may rotate around the screw, potentially leading to the progression of kyphosis. To address this issue, a technique involving the placement of multiple screws in the proximal fracture segment, above the region of kyphosis, was developed. This approach has been shown to enhance stability and prevent further worsening of kyphosis [26].

In cases where pelvic dysmorphism prevents the safe passage of a trans-sacral screw, sacroiliac (SI) screws, which cross only one sacroiliac joint in an oblique cephalad and anterior trajectory, may be used. However, these are often supplemented with a second trans-sacral screw into the S2 vertebral body to achieve more robust fixation [26]. This approach is recommended because trans-sacral screws provide superior biomechanical stability compared to sacroiliac screws [27,28]. Although partially threaded sacroiliac screws with longer threads (32 mm) demonstrate greater pull-out strength than short threaded SI screws (16 mm), trans-sacral screws are particularly effective for managing spinopelvic dissociation, osteoporosis, or nonunion revisions [27,28]. These screws provide superior stability compared to SI screws, as the location of their purchase in the stronger contralateral ilium allows for greater engagement with additional cortical bone [27,28]. The importance of increased fixation is even greater in the geriatric population with poor bone quality, compared to the superior bone quality and improved screw purchase often found in younger patients.

Although trans-sacral screws provide robust fixation of the sacrum to the pelvis, they lack the stability to adequately address spinopelvic dissociation. Pelvic fixation alone is a reasonable option for simple, stable sacral fracture patterns [28]. However, in cases of significant displacement, comminution, kyphosis-inducing horizontal fracture patterns (such as U-type fractures), spinal trauma, or neurologic compromise, lumbopelvic fixation is indicated [28]. The vertical component of U-type fractures is also prone to instability due to vertical shear forces and requires adequate compression for stabilization [28]. Achieving this stability necessitates sufficient bony screw purchase and even force distribution over a long screw, typically through trans-sacral fixation [28]. Although SI screws are an option, they are primarily used for stable SI joint-fracture dislocations with good bone stock, where the joint’s inherent stability supports fixation [28]. They may also be considered in cases of pelvic dysmorphism when trans-sacral screws are not feasible [28]. In cases requiring enhanced rotational and vertical shear control due to significant displacement, comminution, or kyphosis, combining lumbopelvic fixation with trans-sacral screws is recommended [28,29]. Biomechanical studies have demonstrated that triangular osteosynthesis provides the strongest fixation construct for such instances [26,29]. Through recent advancements in MIS techniques, modern fixation constructs, and navigation technology, spinopelvic constructs have evolved to provide robust stability while minimizing complications, offering patients improved outcomes and expedited recovery [7,17,18,19]. Thus, collaboration between spine and orthopedic trauma surgeons is crucial to successfully augment lumbopelvic constructs with trans-sacral screws via triangular osteosynthesis to ensure optimal patient outcomes.

## 5. Techniques and Considerations in Spinopelvic Fixation: Sequence, Reduction, and Avoiding Complications

When determining the sequence of spinopelvic fusion and trans-sacral screw placement, it is generally preferable to place the trans-sacral screw first due to the narrow corridor required for its trajectory. In cases of significant sacral kyphosis where the corridor is obstructed, a closed reduction maneuver may be required before proceeding with trans-sacral fixation. This typically involves prone hyperextension of the hips combined with a posterior-to-anterior directed force over the sacrum [16]. A bump under the thighs may also be used to aid in extension of the hips [9]. If closed reduction methods prove insufficient, percutaneous or open techniques may be necessary. Percutaneous methods, for instance, can involve the use of Schanz pins placed in the L5 pedicles and the PSIS to facilitate fracture reduction [16]. Alternatively, an open midline approach may be employed, wherein a Schanz pin is placed in the upper sacral segment, carefully avoiding nerve roots, and counter-traction is applied using bilateral femoral traction pins [16]. These reduction techniques offer versatile solutions for managing challenging cases of sacral kyphosis that obstruct the corridor required for trans-sacral screw placement.

Avoiding complications in spinopelvic surgery requires adherence to precise techniques. A common medicolegal complaint faced by spine surgeons is wrong-level surgery. Fluoroscopic localization with a spinal needle prior to instrumentation ensures accuracy [30]. Another common potential error of hardware malposition arises from inaccuracies in navigation, often caused by displacement of the reference frame following image acquisition. During open procedures, this can be mitigated when surgeons can confirm observable anatomical landmarks with navigated instruments. In percutaneous MIS procedures, however, this can be significantly more challenging, especially in unstable fracture settings, and surgeons often rely on tactile feedback and experience.

Pedicle perforations during screw placement pose risks to the intervertebral disc, nerve roots, spinal cord, and paraspinal musculature, highlighting the importance of careful intraoperative navigation and neuromonitoring. Real-time neuromonitoring after each screw placement should be employed to identify and correct potential neurologic changes in real time throughout the case. Using robotic techniques may also help reduce the risk of screw breach and angular deviation, as studies have shown a higher proportion of accurately placed screws with robot-assisted techniques compared to freehand techniques [31,32,33].

## 6. Spinopelvic Fixation Percutaneous Technique

**Figure 1:**
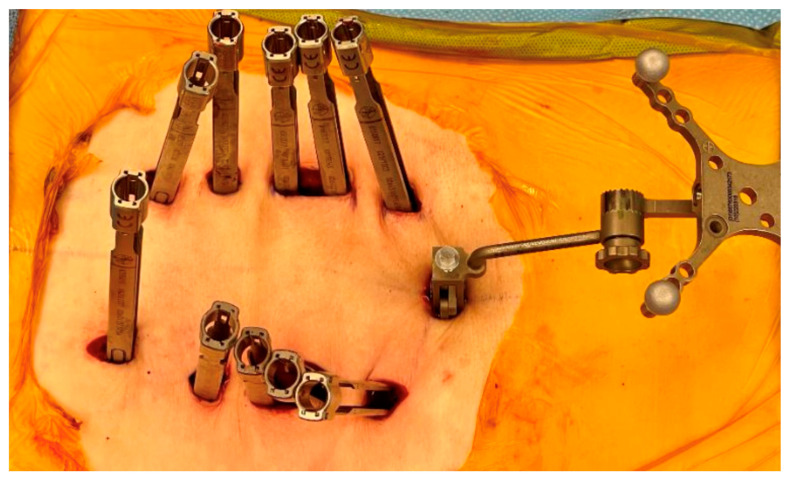
Percutaneous incisions allowing placement of CT reference array and pedicle screw guides.

After confirming the correct operative levels with a spinal needle under fluoroscopy, a mini midline incision is made directly over the most proximal lumbar spine level down to the spinous process. The reference array is then placed and secured tightly. Following image acquisition with intraoperative CT and calibration of the navigation surgical tools, bilateral percutaneous stab incisions are made, followed by insertion of high-speed burr, tap, and subsequent pedicle screws. Following satisfactory hardware position, subfascial rods are passed along the screw tabs to achieve construct stability (Figure 1).

**Figure 2:**
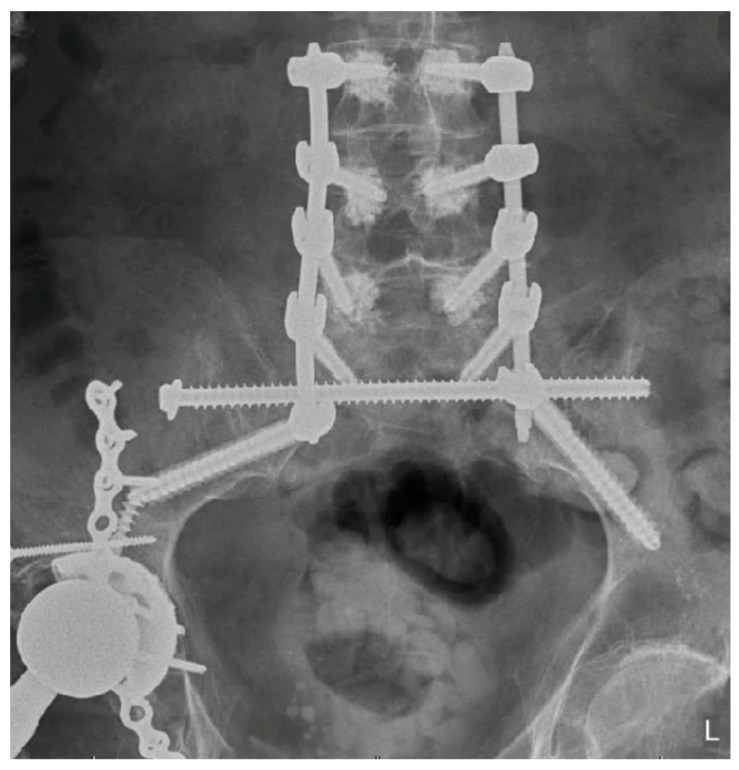
AP Radiograph of cement augmented spinopelvic fusion.

**Figure 3:**
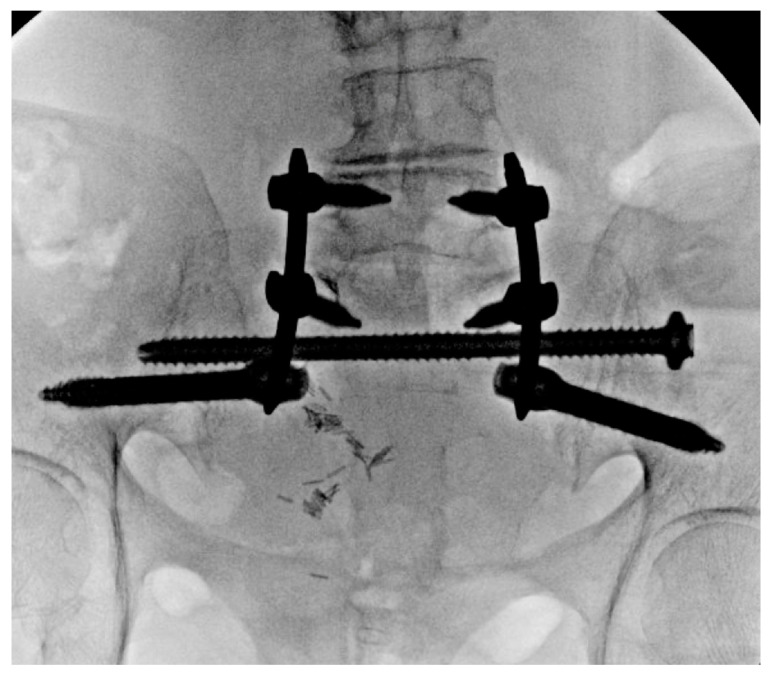
Intraoperative AP fluoroscopy of final lumbopelvic construct augmented with trans-sacral screw fixation via triangular osteosynthesis.

**Figure 4:**
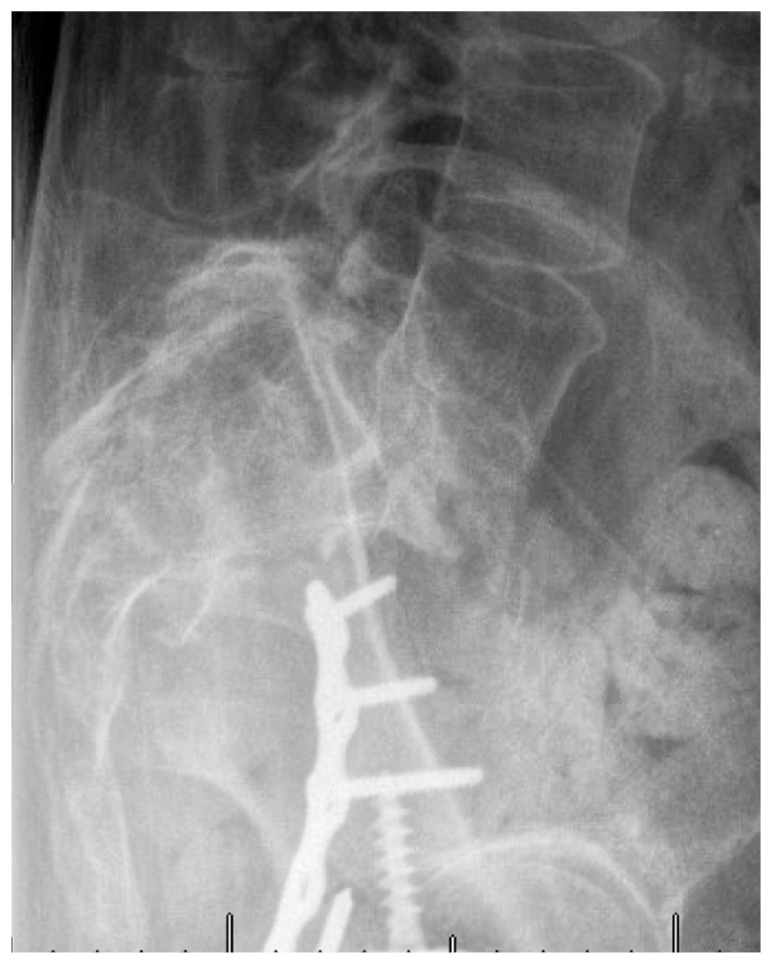
Pre-operative lateral x-ray demonstrating L5-S1 spondylolisthesis and sacral kyphosis.

**Figure 5:**
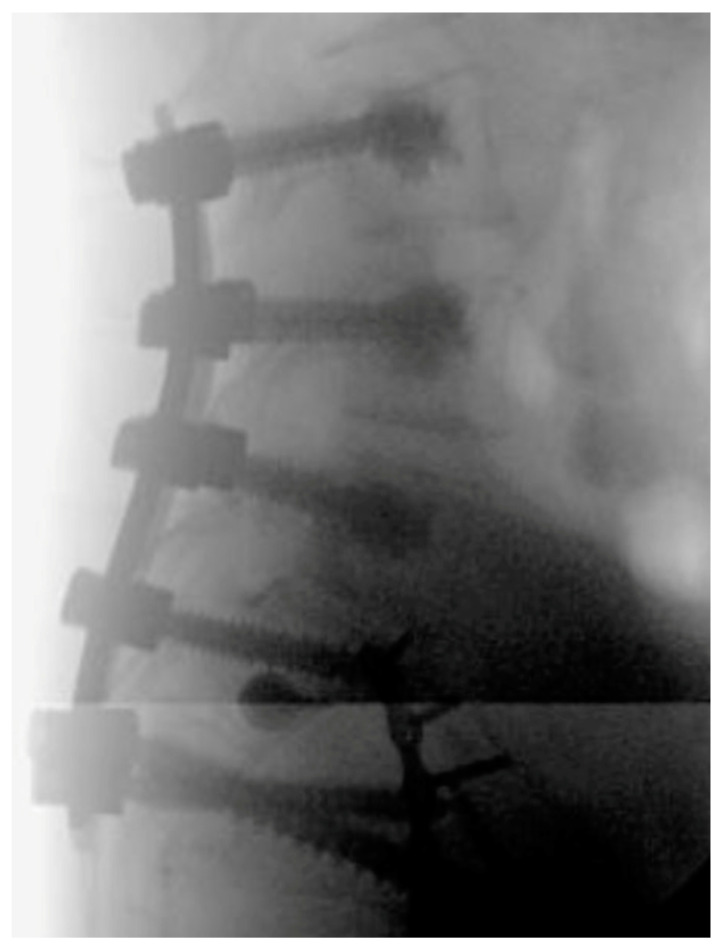
Intraoperative lateral fluoroscopic image of spinopelvic fusion with L5 pedicle screws advanced into the vertebral body enable reduction of L5-S1 spondylolisthesis.

Patients with poor bone quality benefit from techniques that optimize fixation, reduce screw pullout and construct failure. Cement augmentation with fenestrated screws has the potential to double pullout strength and reduce displacement, effectively compensating for severe osteoporosis (Figure 2) [34]. In contrast, cement augmentation is not necessary when there is adequate bone quality (Figure 3). Similarly, cement is avoided in cases of infection due to the risk of retained cement acting as a potential nidus for recurrent infection. In these cases, upsizing the pedicle screw may be employed for improved fixation by achieving higher cortical contact. Another important consideration to reduce construct stress and mitigate the risk of failure is the proper restoration of lumbar lordosis, alignment and spondylolisthesis (Figure 4 and Figure 5), which is often present in the geriatric population due to age-related degeneration. When these patients sustain fractures, the spondylolisthesis may be addressed simultaneously during the procedure for additional construct stability and to achieve fusion.

## 7. Tran-Sacral Percutaneous Technique

**Figure 6:**
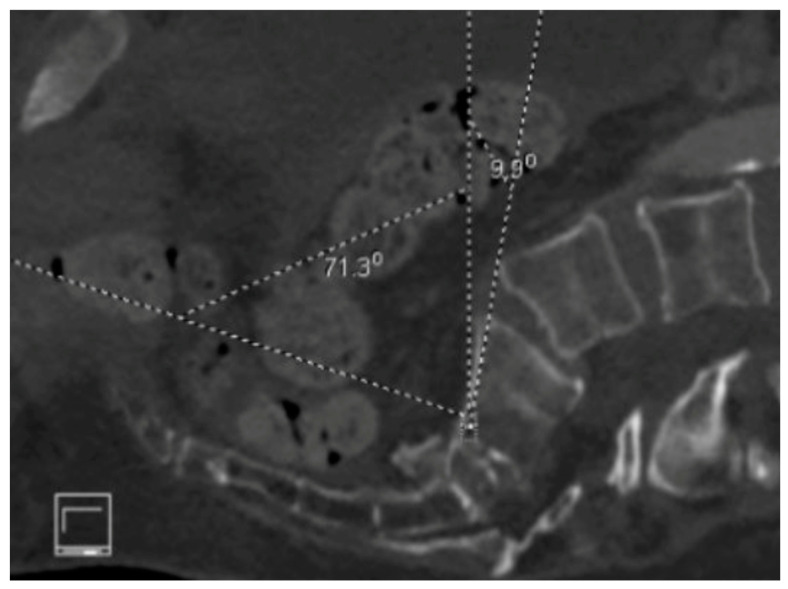
Preoperative Sagittal CT scan demonstrating significant sacral kyphosis and extreme outlet angle.

**Figure 7:**
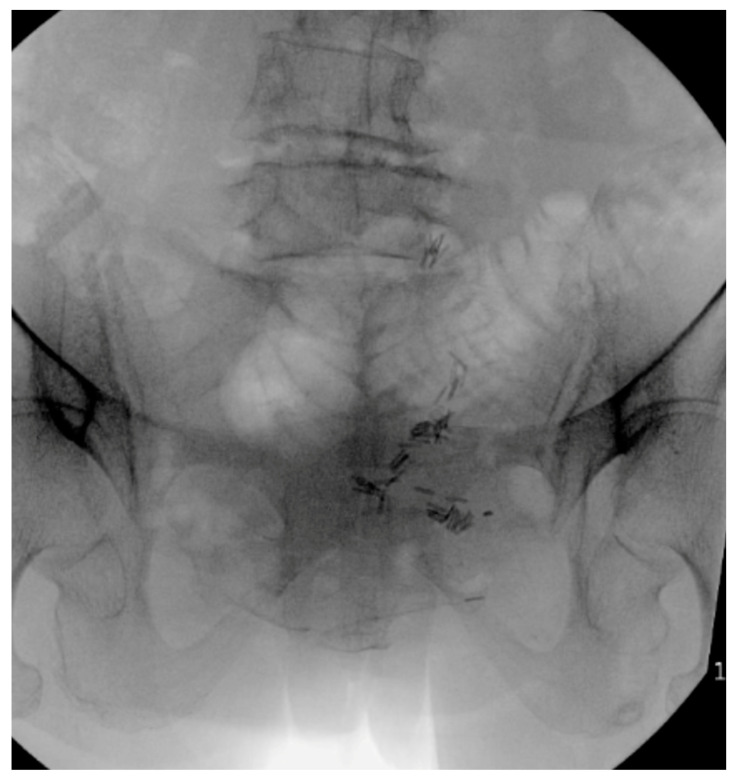
Intraoperative outlet fluoroscopic film with obstructed view of the sacral neural foramina due to bowel gas.

**Figure 8:**
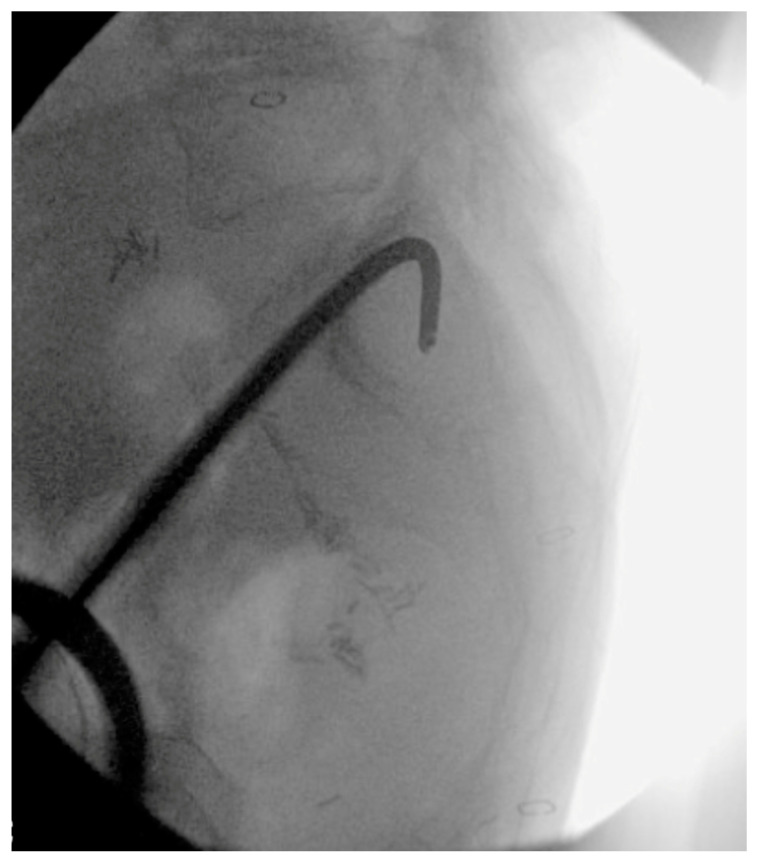
Intraoperative lateral sacral fluoroscopic view with starting wire.

Trans-sacral screws are typically placed in the supine position, which facilitates consistent imaging angles. When supine positioning does not significantly extend anesthesia time, it is a practical choice. However, prone positioning to reduce repositioning, re-prepping, and re-draping time is also feasible, though the trauma surgeon will have to reorient and redirect the C-arm in reverse to obtain the appropriate inlet and outlet views. In these cases, the preoperative CT, performed in supine position, may not perfectly align with intraoperative conditions due to gravitational shifts in pelvic orientation. Significant sacral kyphosis and an extreme outlet angle may make it challenging to achieve the outlet view due to physical obstruction of the C-arm by the patient’s thighs (Figure 6). However, positioning the pelvis on sacral bone foam may reduce the outlet angle, enabling successful visualization of the outlet view. Excessive bowel gas may also hinder adequate visualization using fluoroscopy (Figure 7). When the outlet view is unattainable or obstructed by bowel gas, lateral sacral views (Figure 8) can provide a reliable alternative for accurately assessing superior–inferior screw placement.

**Figure 9:**
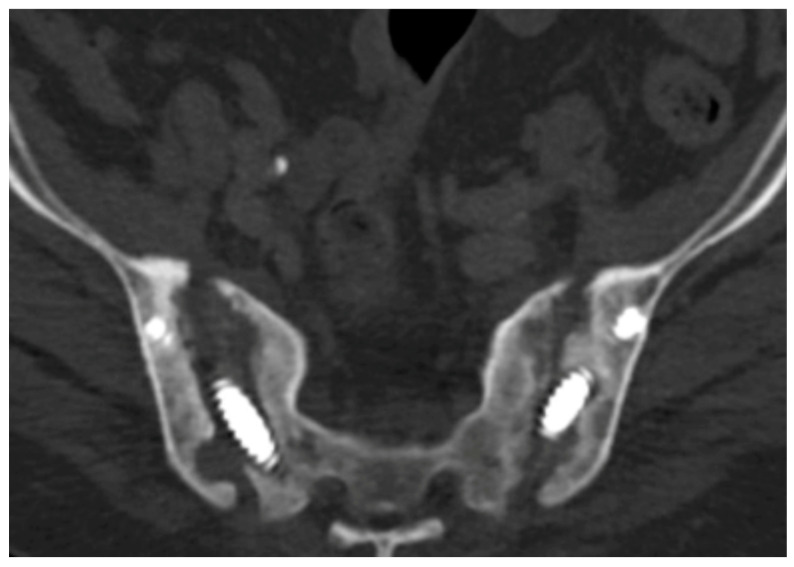
Axial CT view of the pelvis showing bilateral SI joint erosion and previous hardware.

Optimal placement of S1 trans-sacral screws is in the anterior inferior aspect of the S1 vertebral body, which allows for the placement of superior S1 pedicle screws as part of the spinopelvic construct. This configuration biomechanically optimizes fixation and prevents rotational instability around the trans-sacral screw. In cases of U-type sacral fractures not treated with spinopelvic fusion, a second trans-sacral S1 screw can function as an anti-rotation mechanism. In contrast, an additional S2 screw may be more effective for pelvic instability caused by bilateral SI joint erosion (Figure 9).

## 8. Discussion

Open spinopelvic surgery places a significant burden on this patient population, without any conferred improvement in radiographic outcomes or union rates compared to minimally invasive techniques [7]. The choice of surgical technique plays a critical role in patient outcomes, with modern MIS approaches offering several advantages over traditional open procedures. Although literature directly comparing MIS and open methods for treating spinopelvic dissociation is limited, previous studies have demonstrated that MIS techniques, which utilize small incisions, are associated with reduced morbidity. Liu et al. conducted a retrospective review comparing triangular osteosynthesis using robotic MIS versus traditional open approaches for spinopelvic dissociation [35]. The study included 32 patients with spinopelvic dissociation injuries and identified statistically significant differences between the MIS and open groups, both intraoperatively and postoperatively [35]. In the MIS group, the authors reported a significantly reduced mean blood loss of 142.5 mL compared to 612.5 mL (429.8% greater) in the open group (*p* < 0.001) [35]. This finding aligns with results from Pearson et al., who analyzed 31 cases of spinopelvic dissociation treated with either MIS or open lumbopelvic fixation without trans-sacral or iliosacral screws [9]. Pearson et al. found blood loss of 171 mL in the MIS group compared to 538 mL (314.6% greater) in the open group (*p* = 0.0013) [9]. Reduced blood loss lowers the risk of anemia and decreases the likelihood of requiring transfusions. MIS techniques also minimize the potential for hemodynamic instability, which may necessitate additional fluid resuscitation or vasopressors.

Liu et al. also observed shorter surgical times in the MIS group, averaging 148.3 min compared to 185 min (124.5% greater) in the open group (*p* = 0.034) [35]. In contrast, Pearson et al. reported no significant difference in operative time between the two approaches [9]. This discrepancy is likely attributable to the use of robotic navigation in Liu et al.’s study, whereas Pearson et al. relied on fluoroscopy, which may have contributed to longer surgical times. Nevertheless, shorter operative times with MIS navigation techniques help reduce the physiological strain of prolonged anesthesia, an especially important consideration for geriatric patients.

Significant differences were also observed in the postoperative period. Liu et al. reported a shorter average hospitalization duration for the MIS group, with stays averaging 19.9 days compared to 28.6 days (143.7% greater) in the open group (*p* = 0.010) [35]. Shorter hospital stays are advantageous in reducing the risks associated with prolonged hospitalization, such as delirium and hospital-associated infections [19,36]. Pearson et al., however, found no difference in hospitalization duration between the two groups [9]. The authors attribute this to the fact that their percutaneous group had a higher incidence of associated traumatic injuries compared to the open group, including extremity, pelvic, and acetabular fractures, as well as thoracic, abdominal, and spine injuries. These additional injuries may have significantly prolonged hospital stays in the percutaneous group. Extrapolating from their results, one can make the argument that applying MIS techniques in the setting of a polytrauma patient may be beneficial and help improve morbidity and mortality, as these patients are often undergoing multiple other surgeries for various injuries such as long bone fractures.

In terms of fracture healing, Liu et al. found that sacral fractures healed faster in the MIS group, with a mean healing time of 3.8 months compared to 4.7 months in the open group [35]. The use of smaller incisions with reduced paraspinal injury, along with faster osseous healing, likely contributed to the significantly higher Majeed scores observed in the MIS group (87.2) compared to the open group (83.1) [35]. These data suggest that patients undergoing MIS following fracture fixation recover faster with reduced pain.

Liu et al. also identified other benefits of MIS. Fluoroscopy frequency was significantly lower in the MIS group, with an average of 35.4 exposures compared to 45.5 in the open group [35]. This reduction minimizes the cancer risk for both patients and operating room staff, as the risk is directly proportional to the radiation dose [37]. Additionally, drilling time was significantly reduced in the MIS group, averaging 7.1 min compared to 9.6 min in the open group [35]. Reduced drilling time prevents excessive drilling, which can compromise the holding strength of screws and increase the risk of implant failure [35].

**Figure 10:**
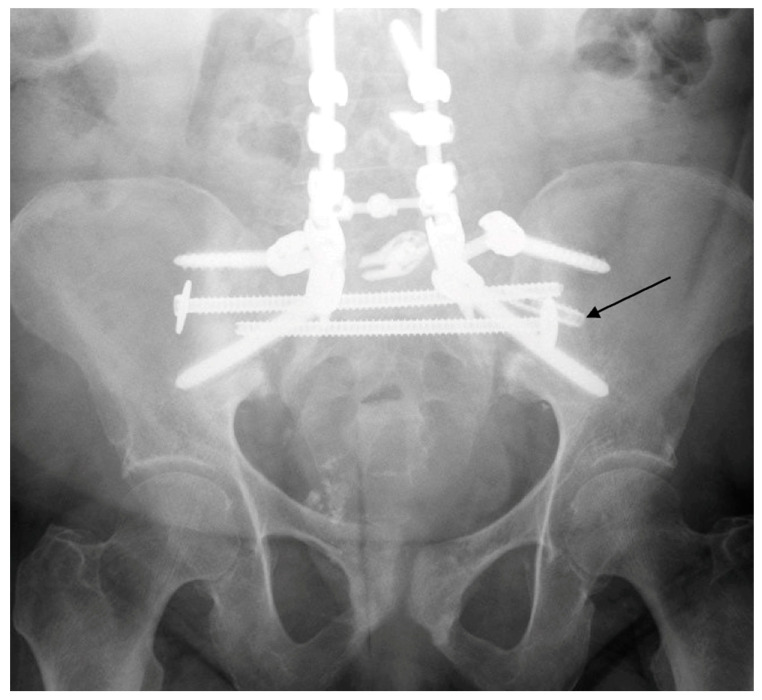
AP radiograph of revision spinopelvic fusion with retained broken screw from previous construct.

MIS techniques, when integrated with advanced technologies, provide additional benefits that are especially valuable in complex and challenging cases. Intraoperative navigation proves indispensable for maneuvering around previously placed hardware, such as acetabular screws (Figure 2 and Figure 4), broken hardware (Figure 10), or trans-sacral screws (Figure 2, Figure 3 and Figure 10). Studies, including those by Shillingford et al., have shown that S2AI screw placement using robotic or navigated freehand techniques achieves comparable outcomes when performed by experienced surgeons [38]. Robotic systems enhance preoperative planning and provide exceptional precision, particularly in cases involving deformity or altered anatomy, and they may improve outcomes for less experienced surgeons [11]. However, robotic technology has limitations, such as constraints on screw depth, which can reduce distal accuracy. There are also complications associated with the learning curve in MIS spine surgery, with an 11% complication rate, including durotomy, implant malposition, neural injury, and nonunion [39]. MIS techniques may also be impractical for significantly displaced, unstable fractures that require open reduction or for cases involving neurological compromise that necessitate open decompression. Finally, navigation technology and robotics increase initial surgical costs, which may not be feasible for certain hospital systems [40]. However, long-term costs for MIS techniques may be lower due to decreased revision rate and infection compared to open techniques [41]. Although MIS techniques offer significant short-term benefits in spinopelvic dissociation, their long-term outcomes for this fracture pattern remain poorly defined. However, in spine surgery for other pathologies, such as degenerative disease, MIS techniques have demonstrated comparable results to traditional open approaches [42].

Despite these challenges, the integration of MIS navigation and robotic techniques offers significant short-term benefits and reduces revision rates in the management of complex spinal cases. However, long-term outcomes of the MIS technique may be comparable to traditional approaches.

## 9. Conclusions

MIS techniques in spinopelvic fixation enable faster recovery, reduced blood loss, and fewer complications requiring revision compared to traditional open methods. However, MIS navigation and robotic surgery have limitations regarding initial cost and indications for use. They are not recommended for significantly unstable, displaced fractures or cases requiring extensive decompression. Through collaboration between spine and orthopedic trauma surgeons, augmentation of spinopelvic constructs with trans-sacral screws via triangular osteosynthesis can result in excellent spinopelvic fixation, critical to the management of patients with spinopelvic instability due to trauma or hardware failure. This collaborative effort coupled with a minimally invasive approach can provide excellent construct stability while minimizing surgical morbidity and complications in patients indicated for spinopelvic fixation.

## Data Availability

No new data were created or analyzed in this study. Data sharing is not applicable to this article.

## Abbreviations

MIS	Minimally Invasive Surgery
PSIS	Posterior Superior Iliac Spine
S2AI	S2-Alar-Iliac
SI	Sacroiliac
IRB	Institutional Review Board
